# Structural, Metabolic and Evolutionary Comparison of Bacterial Endospore and Exospore Formation

**DOI:** 10.3389/fmicb.2021.630573

**Published:** 2021-03-09

**Authors:** Polina Beskrovnaya, Danielle L. Sexton, Mona Golmohammadzadeh, Ameena Hashimi, Elitza I. Tocheva

**Affiliations:** Department of Microbiology and Immunology, Life Sciences Institute, Health Sciences Mall, The University of British Columbia, Vancouver, BC, Canada

**Keywords:** Firmicutes, Actinobacteria, endospore, exospore, regulation, structural biology, bacterial evolution

## Abstract

Sporulation is a specialized developmental program employed by a diverse set of bacteria which culminates in the formation of dormant cells displaying increased resilience to stressors. This represents a major survival strategy for bacteria facing harsh environmental conditions, including nutrient limitation, heat, desiccation, and exposure to antimicrobial compounds. Through dispersal to new environments via biotic or abiotic factors, sporulation provides a means for disseminating genetic material and promotes encounters with preferable environments thus promoting environmental selection. Several types of bacterial sporulation have been characterized, each involving numerous morphological changes regulated and performed by non-homologous pathways. Despite their likely independent evolutionary origins, all known modes of sporulation are typically triggered by limited nutrients and require extensive membrane and peptidoglycan remodeling. While distinct modes of sporulation have been observed in diverse species, two major types are at the forefront of understanding the role of sporulation in human health, and microbial population dynamics and survival. Here, we outline endospore and exospore formation by members of the phyla Firmicutes and Actinobacteria, respectively. Using recent advances in molecular and structural biology, we point to the regulatory, genetic, and morphological differences unique to endo- and exospore formation, discuss shared characteristics that contribute to the enhanced environmental survival of spores and, finally, cover the evolutionary aspects of sporulation that contribute to bacterial species diversification.

## Introduction

Bacteria utilize diverse adaptations for perseverance and propagation under adverse environmental conditions: one such adaptive mechanism is sporulation. The process is characterized by enhanced resilience to stressors, which allows for preservation of genetic material for prolonged periods of time. Sporulation occurs through various modes, many of which remain to be thoroughly investigated and characterized, such as production of akinetes by Cyanobacteria, budding spores within the phylum Chloroflexi, and fruiting body formation in myxobacteria (Proteobacteria) (Kaplan-Levy et al., 2010; Yabe et al., 2010; Muñoz-Dorado et al., 2016). However, the two most extensively studied modes of sporulation are performed by members of the phyla Firmicutes (endospore formation) and Actinobacteria (exospore formation). Here, we discuss their main mechanisms of gene regulation, metabolic and structural transformations, evolutionary significance in the diversity of current bacterial species, as well as outline their shared and contrasting characteristics.

**FIGURE 1:**
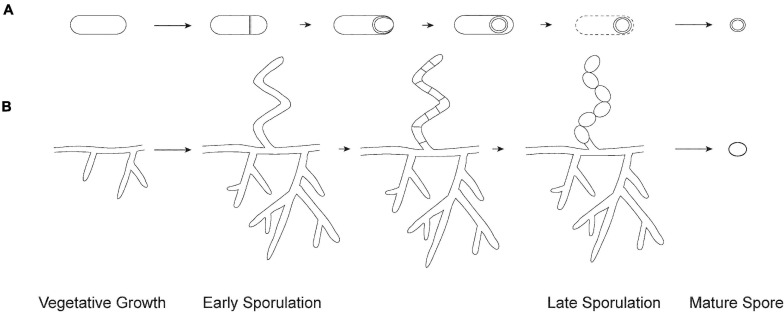
Overview of sporulation. **(A)** Endospore formation in Firmicutes. The process begins with the formation of an asymmetric septum. Next, the larger compartment engulfs the smaller immature spore. The spore matures by the formation of protective layers and is released through lysis of the mother cell. **(B)** Exospore formation in Streptomyces. The process begins by aerial hyphae formation, which subsequently divide into numerous compartments. Each compartment matures into an exospore that gets released from the spore chain.

Bacteria of the phylum Firmicutes have been traditionally distinguished by possession of a low-G + C genome, Gram-positive (monoderm) cell envelope comprising a cytoplasmic membrane (CM) surrounded by a thick layer of peptidoglycan (PG). With recent developments in genome-based taxonomy, this notion has been challenged by the discovery of Firmicutes with relatively high-G + C genomes (e.g., *Geobacillus*), as well as Gram-negative, or diderm, cell envelopes comprising an inner membrane (IM), a thin layer of PG, and a typical outer membrane (OM, e.g., *Acetonema longum*, class Negativicutes) (Yutin and Galperin, 2013). Unsurprisingly, as a result of such variation, Firmicutes have been successful in populating diverse environments, such as soil, ocean and sediments, and colonize various hosts, including insects and mammals. Many Firmicutes are also major human pathogens, e.g., *Clostridium difficile*, *Bacillus anthracis*, and *Streptococcus pneumoniae*. Some Firmicutes, notably within the classes Bacilli and Clostridia, are capable of endospore formation, a process that is restricted to the phylum (Figure 1A) (Beskrovnaya et al., 2020). Endospore formation is characterized by asymmetric cell division of the mother cell, which results in the formation of a smaller compartment (prespore or forespore) that is then engulfed in a phagocytosis-like manner (Tan and Ramamurthi, 2014). The prespore undergoes further maturation through modifications in the cell envelope and the gradual halting of metabolism, ultimately followed by release into the environment with lysis of the mother cell. Endospores are highly resilient dormant life forms that can withstand exposure to extreme heat, desiccation, ultraviolet radiation and other factors, highlighting their significance in pathogenesis (Table 1) (Setlow, 2014).

**TABLE 1:** Effects of stressors on survival of endospores and exospores.

Stressor	Endospore	Exospore
Wet heat (60°C)	Not determined	60% survival after 10 min (Haiser et al., 2009)
Wet heat (80°C)	50% survival after 30 h (Fox and Eder, 1969)	0% survival after 10 min (Kawamoto et al., 1981)
UV 222 nm	10% survival with 250 J/m^2^ (Clauss, 2006)	10% survival with 127 J/m^2^ (Clauss, 2006)
UV 254 nm	10% survival with 520 J/m^2^ (Clauss, 2006)	10% survival with 85 J/m^2^ (Clauss, 2006)
Desiccation at 0% humidity	No effect on viability (Setlow, 2014)	23% survival after 28 days at 25°C (McBride and Ensign, 1987)
Triple autoclaving (heat and pressure)	Survival at 121°C for 20 min, (O’Sullivan et al., 2015)	Not determined

The Actinobacteria (high-G + C genomes) is another diverse phylum, including organisms with a variety of cell morphologies – from cocci and rods to more complex morphologies, such as differentiated mycelia. These bacteria live in a variety of environments, ranging from soil and marine environments to human skin, occupied by commensal organisms. A number of Actinobacteria are human, animal, or plant pathogens as well. Unlike in the Firmicutes, sporulation in Actinobacteria occurs by formation of exospores: new cells are produced through cell division, which then undergo morphological differentiation into mature spores (Hoskisson and Van Wezel, 2019). Speciation within the Actinobacteria has led to a wide variety of exospore formation modes, from single spores produced on undifferentiated mycelia, to the formation of sporangia. The best characterized form of sporulation within the Actinobacteria, however, is the long chains of exospores produced by the soil and ocean dwelling *Streptomyces* (McCormick and Flardh, 2012). *Streptomyces* grows vegetatively by tip extension and branching (Figure 1B). Specialized aerial hyphae extend upwards off the colony surface and undergo synchronous cell division to convert the multinucleate aerial hyphae into dozens of unigenomic spores (Szafran et al., 2020). These spores undergo maturation, which involves thickening of the spore wall and then entry into dormancy. Finally, exospores are presumably released from the spore chain by mechanical means. As is the case with endospores, exospores remain dormant in the environment until favorable growth conditions are sensed, at which point they germinate and resume vegetative growth (Sexton and Tocheva, 2020). Despite being a model system, less is known about the regulation and mechanics of sporulation in *Streptomyces* compared to Firmicutes.

## Gene Regulation

The required set of environmental stimuli needed for initiating sporulation have eluded researchers for years. Generally, the main trigger for initiation of endo- and exospore formation appears to be depletion of nutrients – particularly of readily available carbon, nitrogen, and phosphorus sources (Higgins and Dworkin, 2012; McCormick and Flardh, 2012). However, individual species develop additional mechanisms for detection of niche-specific stimuli, such as exposure to oxygen in obligately anaerobic Firmicutes (Mearls et al., 2012). Although the environmental stimuli for sporulation remain somewhat comparable between Firmicutes and Actinobacteria, gene regulation is vastly different between the two phyla. Because sporulation involves extensive morphological transformations through the coordinated action of hundreds of proteins, it is a highly energetically costly process. Therefore, bacteria have evolved mechanisms to carefully monitor their environment and ensure the appropriate timing of each stage of the process.

### Spo0A Is the Master Regulator in Activation of Endospore Formation

In Firmicutes, the ubiquitously distributed regulator protein Spo0A is recognized as the hallmark component of stress-activated gene expression machinery and, by extension, endospore formation (Ramos-Silva et al., 2019). Currently, over 80 genes, including *spo0A*, are known to be required for endospore formation (Galperin et al., 2021). Consistently, loss of *spo0A* from the genome correlates with the inability to sporulate across the phylum (Galperin et al., 2012). The Spo0A protein comprises two domains: a DNA-binding regulatory domain harboring a characteristic helix-turn-helix motif, and a sensory domain which accepts signal input through phosphorylation of a conserved aspartate residue by a cognate sensor kinase (Zhao et al., 2002). Several cell surface-associated and intracellular histidine kinases (Kin) implicated in direct and indirect phosphorylation of Spo0A have been identified in Bacilli and Clostridia (Jiang et al., 2000; Underwood et al., 2009; Steiner et al., 2011).

Unsurprisingly, despite possessing the structural and sequence features characteristic of histidine kinases, variability within the ligand-binding sites of their sensor domains makes it difficult to predict the exact signals recognized by each enzyme (Fabret et al., 1999). Therefore, the specific triggers and receptors that contribute to phosphorylation of Spo0A are not universally conserved between endospore formers. In *Bacillus subtilis*, for example, upon sensing extra- or intracellular signals, Kin proteins transfer a phosphoryl group onto the response regulator Spo0F, which in turn transfers the phosphate to the phosphotransferase Spo0B, which, lastly, phosphorylates Spo0A. Additionally, phosphatases present at each step in the phosphorelay system aid in finer regulation of phosphorylated levels of each protein and, ultimately, Spo0A (Fabret et al., 1999). In contrast, Clostridia, while relying on the same mechanism, make use of a simpler system lacking the intermediate components between Kin proteins and Spo0A (Stephenson and Hoch, 2002).

Elevated levels of Spo0A∼P are required for sporulation to occur (Fujita et al., 2005). Stochasticity is presumed to play a major role in driving the intracellular concentration of Spo0A∼P toward the level required for sporulation to commence (Maamar et al., 2007; Chastanet et al., 2010; de Jong et al., 2010). However, other endogenous stress response pathways that are similarly stimulated by the presence of Spo0A∼P simultaneously compete for control over cellular processes. This includes competence, biofilm formation and cannibalism (Maamar et al., 2007; Chastanet et al., 2010; de Jong et al., 2010). Here, cell fate is determined by the outcomes of co-occurring processes that indirectly affect phosphorylation of Spo0A at transcriptional, translational and post-translational levels.

### Second Messenger c-di-GMP Regulates Major Checkpoints in *Streptomyces* Development

Rather than using a master regulator such as Spo0A, sporulation in *Streptomyces* is extensively regulated by levels of the nucleotide second messenger c-di-GMP. c-di-GMP is a cyclic dimer of two GMP molecules produced by diguanylate cyclases and degraded by phosphodiesterases containing EAL domains. While the signals regulating the activity of diguanylate cyclases and phosphodiesterases are unknown, the phenotypes associated with deletion of individual enzymes are different, suggesting that they respond to distinct environmental cues (Haist et al., 2020). Intracellular levels of c-di-GMP direct passage through two checkpoints in *Streptomyces* sporulation. The first is the initial production of sporulative hyphae, under the control of the master regulator of sporulation, BldD. Dimers of BldD are held together by a tetramer of c-di-GMP, allowing BldD to bind DNA to repress transcription of various genes involved in sporulation (Tschowri et al., 2014). It is currently unknown what causes the dissociation of BldD-c-di-GMP complexes to initiate sporulation; however, this likely involves a reduction in the concentration of intracellular c-di-GMP. Following the relief of transcriptional repression, the sigma factors σ^BldN^, σ^H^, and σ^WhiG^ are produced. These sigma factors regulate, either directly or indirectly through transcription factors, the expression of genes critical for the formation of aerial hyphae, as well as genes involved in cell division, chromosome segregation and condensation, and spore maturation. The second major checkpoint in sporulation is regulation of σ^WhiG^, responsible for directing transcription of genes involved in chromosome segregation, cell division, spore maturation, and chromosome compaction (McCormick and Flardh, 2012). σ^WhiG^ is regulated by its cognate anti-sigma factor RsiG in a c-di-GMP dependent manner (Gallagher et al., 2020). High intracellular concentrations of c-di-GMP promote the formation of a heterodimer of σ^WhiG^ and RsiG, preventing σ^WhiG^ from interacting with RNA polymerase (Gallagher et al., 2020). When the concentration of c-di-GMP is reduced, the σ^WhiG^ and RsiG heterodimer dissociates, allowing σ^WhiG^ to bind to RNA polymerase (Gallagher et al., 2020). Similar to regulation by BldD, it is unknown how the concentration of c-di-GMP in the cell is reduced. Intriguingly, c-di-GMP is also used to regulate morphological differentiation in *B. subtilis*; however, it controls the transition from motility to biofilm formation (Chen et al., 2012).

## Entry Into Sporulation

### Cell Division and Cell Envelope Transformation During Sporulation

**FIGURE 2:**
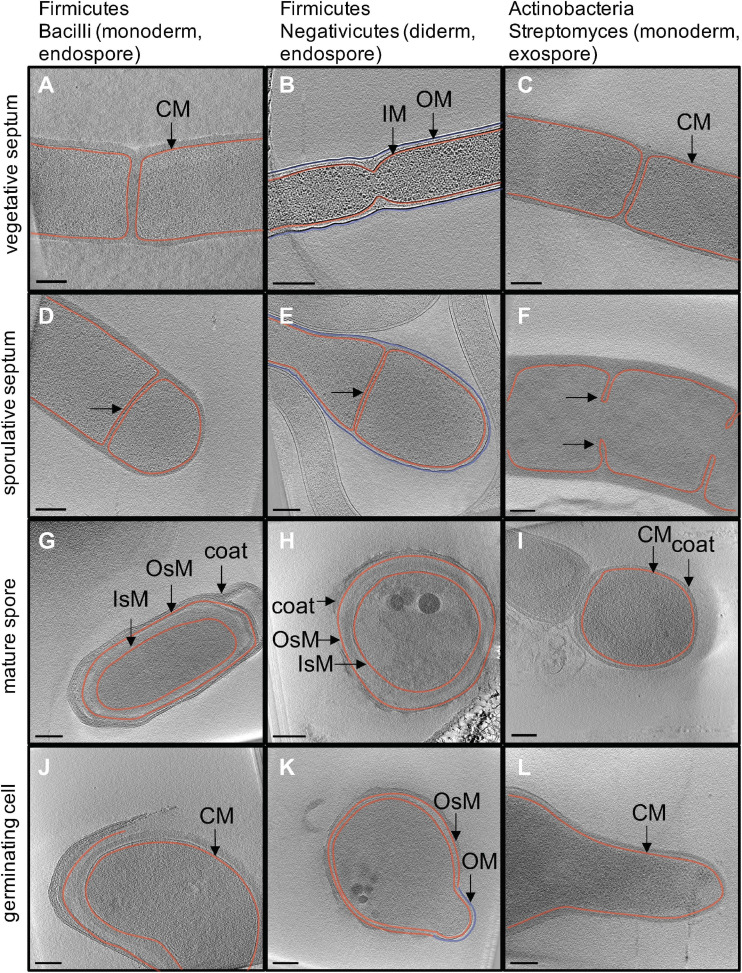
*In situ* structural detail of sporulation revealed by cryo electron tomography. Each image is a 20-nm slice through a tomogram. Column one **(A,D,G,J)** outlines major stages of endospore formation in the model organism *Bacillus subtilis*. Column two **(B,E,H,K)** outlines endospore formation in *Acetonema longum*, a diderm Firmicute and member of the Negativicutes. Column three **(C,F,I,L)** outlines exospore formation in *Streptomyces albus*. Cytoplasmic (CM) and inner membranes (IM) are shown in red, outer membrane (OM) is shown in blue. The inner and outer spore membranes in Firmicutes (IsM and OsM) are both colored in red to show that they are derived from the CM/IM of the mother cell.

During vegetative growth, bacteria divide through a process called binary fission, where the cell divides symmetrically. Typically, this results in the formation of two daughter cells that are identical to the mother cell. Thus, vegetative septa in Firmicutes and Actinobacteria are formed by the pinching of the cytoplasmic membrane (CM in monoderm cells, Figures 2A,C), or the inner and outer membranes (IM and OM in diderm cells, Figure 2B). Alternatively, sporulation provides a different mode of cell division. In Firmicutes, the mother cell undergoes asymmetric division, generating two compartments of different sizes. The bigger one is the mother cell, whereas the smaller one becomes the mature endospore (Figures 1, 2D,E). Notably, sporulative septa in diderm bacteria are formed by invagination of the IM only (Figure 2E) (Tocheva et al., 2011). High-resolution imaging of thin sections in *B. subtilis* also reveals the presence of thinner septa in sporulating cells (∼23 nm vs ∼80 nm in vegetative cells) (Khanna et al., 2019). Despite the diversity in cell envelope architecture among the sporulating Firmicutes, the subsequent phagocytosis-like engulfment by the mother cell always generates an intracellular immature spore bound by two lipid bilayers, the inner (IsM) and outer (OsM) spore membranes (Figures 2G,H) (Tocheva et al., 2011, 2013) (whereas mature exospores of Actinobacteria are surrounded by one membrane only, Figure 2I). During the early stages of endospore formation, control over gene expression in both compartments is transferred from the master regulator Spo0A to the mother and daughter cell specific sigma factors, σ^F^ and σ^E^, respectively. This transition toward σ^E^-driven gene expression, as well as rapid autoproliferation, signifies the point of irreversible commitment to endospore formation, which coincides with engulfment of the prespore.

At the start of endospore formation, membrane-associated phosphatase SpoIIE, exclusive to endospore formers and previously implicated in septum positioning and thinning (Illing and Errington, 1991; Barák and Youngman, 1996; Khanna et al., 2019), recruits two filamentous cytoskeletal proteins FtsZ and FtsA. Here, FtsZ monomers polymerize into short filaments in a GTP dependent manner. These filaments are bundled together into rings which drive cell division through GTP hydrolysis and treadmilling to constrict the Z ring. Interestingly, unlike in vegetative cell division, FtsAZ filaments are restricted only to the mother cell face of the septum due to association with SpoIIE (Khanna et al., 2019). This pattern of co-localization is presumed to accommodate attachment of additional proteins for cell wall synthesis in early and late endospore formation, including PG remodeling by the SpoIIDMP machinery and formation of the SpoIIQ-SpoIIAH channel complex described further in this review (Bisson-Filho et al., 2017; Squyres et al., 2020).

The spore cortex is a thick layer of modified PG deposited between the IsM and OsM, which differs from vegetative PG by increased thickness and a reduced number of peptide crosslinks (Tocheva et al., 2013; Ojkic et al., 2016). During engulfment, PG remodeling is carried out by the SpoIIDMP complex consisting of two hydrolases, the amidase SpoIID and transglycosylase SpoIIP, as well as the transmembrane scaffolding protein SpoIIM. Both SpoIID and SpoIIP are crucial for this process in *Bacillus* and *Clostridium*, whereas SpoIIM is only required in *Bacillus* (Ribis et al., 2018). Surprisingly, bioinformatics analyses show that while SpoIIM is well-conserved across all endospore formers, SpoIID and SpoIIP demonstrate high primary sequence variability outside the key catalytic residues directly involved in cortex formation (Kelly and Salgado, 2019). Based on the compartmentalization of their respective regulons, DMP is presumed to at least partially anchor on the mother cell side in Bacilli and on the forespore side in Clostridia (Kelly and Salgado, 2019). The fully assembled complex localizes at the septal midpoint and moves to the leading edge of the engulfing membrane. This localization is dependent on SpoIIM in *Bacillus*, but not *Clostridium* (Dembek et al., 2018). Imaging studies in *B. subtilis* additionally demonstrate that during engulfment, septum PG exhibits homogenous thinning, likely due to increased turgor (Khanna et al., 2019). Moreover, cortex formation proceeds through PG synthesis at the leading edges of engulfing membranes, followed by sequential hydrolysis by SpoIIDP (Tocheva et al., 2013; Dembek et al., 2018). As SpoIID removes peptide crosslinks, SpoIIP follows with cleavage of “denuded” glycans leaving NAG-NAM disaccharides. Altogether, this process is suggested to drive membrane engulfment (Tocheva et al., 2013; Khanna et al., 2019). Cortex formation is finalized via activity of SpoV proteins: the putative membrane-associated lipid II flippase SpoVB, and the penicillin-binding proteins SpoVDE, produced by the mother cell (Popham and Stragier, 1991; Vasudevan et al., 2009; Cho et al., 2016; Bukowska-Faniband and Hederstedt, 2017). These proteins are essential for cortex formation and incorporate PG precursors provided by the mother cell. Experimental work in *Bacillus* reveals that absence of SpoVBDE blocks cortex formation and leads to accumulation of PG precursors (Vasudevan et al., 2007). In Clostridia, these proteins are conserved, but it is not known whether they are dispensable.

During engulfment, SpoIIQ, a protein under the control of σ^F^ in the daughter cell, is found throughout the daughter cell lipid membrane. Conversely, SpoIIIAH, a protein under the control of σ^E^ (*spoIIIA* operon) in the mother cell, is found on the mother cell lipid membrane. The two proteins form a complex via direct interactions between their extracellular domains in the intermembrane space, as the septal PG is degraded by the DMP complex. As multiple AH-Q multimers are formed at the interface between the mother and daughter cells, each of them further recruits additional proteins, such as SpoIIIAA-AG (also part of the *spoIIIA* operon), and GerM in *Bacillus* to assemble a multimeric complex (AH-Q channel) connecting the two compartments (Trouve et al., 2018). Although the resulting structure has not been observed directly *in vivo* with high resolution structural approaches, previous studies suggest that it comprises a channel ring, composed of IIIAG, IIIAH and IIQ oligomers, tethered to an ATPase and a basal body, with individual components displaying homology to proteins in type II, III, and IV secretion systems (Fimlaid et al., 2015b). Further, despite identification of the complex in both Bacilli and Clostridia, its structure and functional roles vary across the phylum. As such, cells lacking the channel show varying levels of compromised activity of σ^G^, suggesting that it at least indirectly functions to transport as yet unknown molecules needed for σ^G^ activation prior to and after engulfment (Doan et al., 2009; Fimlaid et al., 2015b; Serrano et al., 2016). It has also been suggested that the complex could transport metabolites and small protein components required for spore maturation from inside the mother cell (Camp and Losick, 2009). The AH-Q channel also appears to play a major structural role, such as in the prevention of membrane deformation and spore collapse during engulfment by tethering of the mother and daughter cells, and in the facilitation of spore coat attachment in Clostridia (Doan et al., 2009; Fimlaid et al., 2015b; Ribis et al., 2018). Upon completion of engulfment, the AH-Q complex is disassembled with the help of the σ^G^-controlled protease SpoIVB (Chiba et al., 2007).

In Firmicutes, septum formation is essential for both vegetative cell division and sporulation. In contrast, while cell division is not essential for *Streptomyces* vegetative growth, it is required for sporulation (McCormick et al., 1994). *Streptomyces* is hypothesized to use a positive selection mechanism to determine septum placement. The first protein that is known to localize to sites of future cell division is SsgA (sporulation of *Streptomyces griseus*). SsgA associates closely with the membrane but is not a membrane protein itself, so how SsgA localization is initially determined remains unknown (Noens et al., 2005; Traag and van Wezel, 2008). Because SsgA is limited to the streptomycetes (Girard et al., 2014), the mechanism for selection of cell division sites in other exospore forming actinomycetes also requires investigation. Following localization to sites of future cell division, SsgA is thought to recruit SsgB, an SsgA-like protein (SALP), which in turn recruits FtsZ (Willemse et al., 2011), which forms Z-rings in an evenly spaced ladder-like pattern along the length of aerial hyphae. Interestingly, *Streptomyces* lacks FtsA, ZipA and Zap proteins which traditionally anchor Z rings to the membrane and enhance Z ring stability. Instead, *Streptomyces* utilizes SepF homologs to anchor Z rings to the membrane during cell division in sporulation (Schlimpert et al., 2017). SepH, an Actinobacterial specific protein, is also integral for establishing the formation of Z rings (Ramos-Léon et al., 2020). However, it is unclear how SepH is initially localized and whether it interacts with other proteins for positioning of the Z rings. Z ring formation is also stabilized by DynA and DynB, two dynamin-like proteins which interact directly with SsgB and SepF2 during division (Schlimpert et al., 2017). PG synthesis and membrane invagination are required to drive cell division. This process is coordinated by FtsL, DivIC, FtsQ, FtsW, FtsI, and FtsEX. While many of these proteins are essential for viability of unicellular bacteria, deletion of these genes has no impact on the viability of *Streptomyces* species. Instead, deletion of these genes result in defects in cell division during sporulation ranging from no apparent effects for FtsE and FtsX (McCormick, 2009), to DivIC, FtsL, FtsW, and FtsI, which only have severe effects on completion of cell division under high osmolarity (Bennett et al., 2007, 2009). While many features of cell division are the same in *Streptomyces* and other unicellular bacteria, mechanisms for determining septum positioning, and the importance of proteins in the divisome to overall viability, are different.

### Chromosome Segregation

During chromosome segregation in Firmicutes, intracellular levels of Spo0A∼P control the activity of DnaA, a bacterial DNA replication protein involved in cell division, via the regulator protein SirA, which also plays a role in polar localization of chromosomes in endospore formation (Jameson and Wilkinson, 2017). Prior to septum formation, Soj and Spo0J proteins tether chromosomes by their oriC region at each pole of the cell (Duan et al., 2016). Soj and Spo0J are homologues of the ParAB system: Soj/ParA is a Walker type ATPase protein which undergoes ATP hydrolysis to translocate foci of Spo0J/ParB bound to the chromosome (Soh et al., 2019; Jalal et al., 2020). Thus, asymmetric division leads to entrapment of one third of the chromosome in the prespore, necessitating utilization of DNA transport machinery for complete segregation. For this purpose, the protein SpoIIIE oligomerizes into a hexameric channel and uses the energy from ATP hydrolysis for DNA transport (Burton et al., 2007; Fiche et al., 2013; Cattoni et al., 2014). Chromosomal DNA then traverses the two lipid membranes into the prespore in a directional manner by binding of non-coding recognition sequences at the SpoIIIE γ domain, resulting in activation of ATPase (Besprozvannaya et al., 2013; Besprozvannaya and Burton, 2014; Bose et al., 2016). Consistently, SpoIIIE shows homology to another type of DNA pump involved in vegetative cell division, FtsK (Besprozvannaya and Burton, 2014). Super-resolution microscopy reveals that SpoIIIE first localizes at the leading edge of the septum, but eventually moves towards the septum midpoint (Fiche et al., 2013). The *in vivo* stoichiometry and mechanism of DNA translocation during endospore formation remain unknown.

In *Streptomyces*, chromosome segregation during sporulation occurs simultaneously with selection of numerous cell division sites. Chromosomes are segregated predominantly using the *parABS* system. ParA is thought to form mitotic spindle like filaments in *S. coelicolor* aerial hyphae during the early stages of segregation (Jakimowicz et al., 2007). ATP hydrolysis by ParA drives translocation of ParB foci into position between forming Z rings (Jakimowicz et al., 2005; Jakimowicz et al., 2007), suggesting that there is an as yet undiscovered interaction between chromosome partitioning and the selection of sites for future cell division. Furthermore, deletion of *parA* affects both chromosome segregation and septum placement (Jakimowicz et al., 2007). ParJ, a protein specific to the Actinobacteria, interacts directly with ParA to regulate its polymerization (Ditkowski et al., 2010). In *par* mutants, almost 90% of spores still contain at least one copy of the chromosome (Kim et al., 2000), suggesting that there are additional mechanisms for chromosome segregation. FtsK, a SpoIIIE homolog, is positioned on the leading edge of the invaginating membrane and may assist with resolving the ends of the linear chromosome to prevent truncation of the arms (Dedrick et al., 2009). *Streptomyces* chromosomes contain additional FtsK-like proteins. One of these, SffA, is localized to closing septa during sporulation by SmeA (Ausmees et al., 2007). Deletion of *sffA* and *smeA* results in a fivefold increase in the number of anucleate spores compared to the wildtype, although it remains unclear if SffA functions as a DNA pump, similar to SpoIIIE, or if it promotes chromosome segregation in a different manner (Ausmees et al., 2007). Links between chromosome segregation and cell division during sporulation are still undefined and offer many exciting avenues for future research.

## Spore Maturation

Endospore maturation begins during prespore engulfment and culminates in lysis-mediated spore release. Here, control over gene expression is gradually transferred to the late-stage sigma factors of the prespore, σ^G^, and the mother cell, σ^K^. Maturation involves finalization of cortex formation, assembly of the protein coat and in some cases, an exosporium, dehydration of the endospore core, and DNA compaction. Altogether, these morphological changes account for immense resistance to biotic and abiotic stressors allowing for preservation of endospores in harsh environments for extended periods of time.

Unlike in endospore formers, engulfment of the spore does not occur in Actinobacteria and the mature spore is bound by one membrane, rather than two (Figure 2I). Following septation (Figure 2F), cells undergo division via rapid mechanical separation, or “V snapping” (Zhou et al., 2016). Immature spores remain associated with each other in the spore chain as they undergo maturation, likely held in place by the rodlet ultrastructure that forms a sheath on the surface of aerial hyphae. Maturation is accompanied by an increase in spore wall thickness (Figure 2I), presumably directed by MreB and other components of the spore wall synthesizing complex, consisting of proteins from the *mre* gene cluster along with RodZ, FtsI, and SCO3901 (Heichlinger et al., 2011; Kleinschnitz et al., 2011; Girard et al., 2014; Sexton and Tocheva, 2020). In addition to increased thickness, the PG in the spore wall features an increase in 3–4 crosslinks and a decrease in 3–3 crosslinks (van der Aart et al., 2018), and multiple layers are visible in the spore wall after spore maturation (Sexton and Tocheva, 2020). It is unclear if the spore wall layers feature the same PG structure, and what the biological significance of modified PG crosslinking is in the spore wall. Several cell wall lytic enzymes, including carboxypeptidase, endopeptidases, and lytic transglycosylases, have been implicated in PG remodeling during exospore maturation (Haiser et al., 2009; Sexton et al., 2015; Rioseras et al., 2016). While these enzymes are critical for spore PG architecture, the modifications they perform and their impact on spore integrity require further investigation.

### Metabolic Adaptations for Environmental Resilience of Spores

Mature endospores are characterized by a unique phase-bright appearance when imaged with phase-contrast light microscopy, owing to their dehydrated state. A higher solids content and lower water content in the spore generate a higher refractive index thus resulting in phase reversion from dark to bright. Dehydration is achieved by several mechanisms. Initially, ATP hydrolysis and loss of K^+^ ions between the mother cell and the spore generate osmotic dehydration. Subsequently, simultaneous replacement of water with dipicolinic acid (DPA) and minerals is thought to decrease hydration even further (Marquis, 1998). DPA is found in all endospore formers, constitutes up to 15% dry weight of mature endospores and has been linked to increased heat resistance (Powell, 1953; Church and Halvorson, 1959; Setlow et al., 2006). Synthesis of DPA occurs in the mother cell under the control of σ^K^ in late stages of sporulation, and is mediated by the enzymes DpaA and DpaB of the *spoVF* operon, through transformation of L-2,3-dihydrodipicolinate, an intermediate of the lysine biosynthesis pathway (Daniel and Errington, 1993; Takahashi et al., 2015). Interestingly, an alternative pathway for DPA synthesis, possibly of ancient origin, has been identified in Clostridia and is performed by the protein EtfA using the product of DpaA activity (Orsburn et al., 2010; Donnelly et al., 2016). DPA relies on a two-step transport pathway: it is taken up by the predicted SpoVV transporter in the OsM for transport into the intermembrane space, then by the multicomponent SpoVA transport system through the IsM (Li et al., 2012; Ramírez-Guadiana et al., 2017). Once inside the spore, DPA associates with calcium ions. Eventually, spore water content decreases to a level that is no longer supportive of unrestricted movement of macromolecules, causing metabolic dormancy.

Similar to endospores, exospores produced by *Streptomyces* species undergo dramatic changes to protect cytoplasmic contents during dormancy. Several proteins and mRNAs are deposited in the spore cytoplasm prior to dormancy for use during germination (Mikulik et al., 2002; Strakova et al., 2013). In order to preserve these proteins and mRNAs, trehalose, a dimer of two glucose molecules joined through an α,α-1,1-glycosidic linkage, is deposited in large aggregates in the spore cytoplasm. Depending on growth conditions, trehalose can comprise up to 25% of the dry spore biomass (McBride and Ensign, 1987). Trehalose interacts with the cytoplasmic contents of the spore through hydrogen bonding, allowing it to protect a wide variety of macromolecules from denaturation and aggregation during periods of excessive dehydration, heat, freezing, radiation and oxidative damage. Additionally, trehalose likely serves as an energy source to be used during the initial stages of germination (McBride and Ensign, 1987). During spore maturation, the spore pigment produced by the *whiE* gene cluster is also deposited inside cells. The exact function of the spore pigment has not yet been determined; however, it seems reasonable to assume that it functions to protect against UV damage. Deletion of the genes responsible for spore pigment in *S. griseus* result in a slight increase in susceptibility to UV irradiation (Funa et al., 2005). Mature *Streptomyces* spores display decreased resistance to a variety of abiotic stressors, including heat and desiccation, when compared to endospores (Table 1). Intriguingly, *Streptomyces* exospores exhibit low level metabolic activity (Constant et al., 2008; Liot and Constant, 2016), which may contribute to reduced tolerance to abiotic stresses in the environment when compared to Firmicutes.

### DNA Compaction

At the end of spore maturation, the chromosome is condensed, likely offering an additional layer of protection for genetic material during dormancy. This is accomplished through the action of nucleoid associated proteins, DNA supercoiling, molecular crowding, and dehydration of the spore. In Firmicutes, dormant endospores exhibit resilience to UV damage due to protection of DNA by DNA-binding proteins called small acid-soluble proteins, or SASPs. SASPs display a range of sizes from 5 to 7 kDa and are produced in the forespore, from *ssp* genes primarily under the control of the late sporulation sigma factor σ^G^ (Setlow, 1988; Wetzel and Fischer, 2015). Evidence suggests that all endospore formers harbor SASPs of the α/β type, whereas small amounts of the non-DNA binding γ SASP have only been identified in *Bacillus* (Setlow, 1988; Vyas et al., 2011). Despite the variety of SASPs produced across all endospore-forming species, each organism displays a major preference toward one or two α/β type SASPs for DNA packaging. SASPs are well-conserved at the DNA-binding helix-turn-helix domains, but can otherwise show sequence variability, particularly between aerobes and anaerobes (Setlow, 2007; Vyas et al., 2011; Wetzel and Fischer, 2015). SASPs restrict access of mutagens to DNA strands by physical protection. However, harmful UV rays, heat and mutagens can still damage DNA while the cell is metabolically dormant. For this, endospores rely on pre-packaged DNA repair enzymes to revert the damage in germination, before cell division resumes (Setlow, 2007).

In *Streptomyces*, chromosome compaction is much more elaborate and involves the action of several classes of nucleoid associated proteins rather than SASPs. SMC complexes promote chromosome compaction and segregation, potentially through an interaction with ParB (Dedrick et al., 2009; Kois et al., 2009). In *S. coelicolor*, this complex has two additional interacting partners: ScpA and ScpB, which both promote chromosome compaction (Dedrick et al., 2009; Kois et al., 2009). sIHF and the HU-like protein HupS function as analogs for histones during sporulation to enhance chromosome compaction (Salerno et al., 2009; Swiercz et al., 2013). DbdA has a histone-like domain which is involved in compacting the DNA, which enhances resistance to oxidative stress (Aldridge et al., 2013). A Dps homolog, DpsA, interacts with the chromosome in a sequence-independent manner to promote compaction. Deletion of *dpsA* results in irregularly long spores which often contain multiple copies of the chromosome, suggesting an interplay between chromosome segregation and septation (Facey et al., 2009). However, *dpsA* is not widely conserved in *Streptomyces* species (Szafran et al., 2020). Dps-like proteins also oxidize iron and deposit ferric oxide, which may protect DNA from oxidative damage, but it is unclear whether other Dps homologs would fulfill this function in other streptomycetes and in Actinobacteria in general. Collectively, these nucleoid-associated proteins condense the chromosome and shield it from damage that may occur during dormancy.

### Assembly and Synthesis of the Spore Coat and Exosporium

The endospore coat is a multilayer protein structure that encapsulates the OsM and provides protection from lytic enzymes, heat, physical disruption, UV radiation, predation and other factors (Figures 2G,H) (Riesenman and Nicholson, 2000; Klobutcher et al., 2006; Setlow, 2006; Alves Feliciano et al., 2019). The coat is a highly complex structure made up of dozens of proteins, many of which are unique to individual species. For example, the *B. subtilis* spore coat is made up of over 70 proteins, but only a small fraction of them have orthologs in other Firmicutes (Abhyankar et al., 2013). The majority of these conserved proteins are morphogenic, meaning that they play important roles in the initial assembly of the coat. One such example of a morphogenic protein is SpoVM, a 3 kDa-sized amphipathic helix produced shortly upon completion of septum formation and during engulfment, which is under the control of σ^E^ in the mother cell. SpoVM is driven to the OsM due to its increased preference for lipid membranes with increased curvature (Levin et al., 1993; Kim et al., 2017). Here, it presumably utilizes a “dash-and-recruit” mechanism of rapid accumulation of SpoVM monomers and their subsequent polymerization (Kim et al., 2017). Then, SpoIVA attaches to SpoVM and mediates assembly of the coat base layer, or “platform,” through ATP hydrolysis. Experimental evidence shows that while both of these proteins are essential for coat assembly in *Bacillus*, in Clostridia the vital role of SpoVM is diminished and instead carried out by the functionally homologous SipL (Stevens et al., 1992; Levin et al., 1993; Ribis et al., 2017). Intriguingly, deficiencies in these coat platform proteins also inhibit formation of the cortex (Ebmeier et al., 2012; Benito de la Puebla et al., 2020). Additional spore coat morphogenesis proteins of Bacilli include SafA and CotE, which are essential for formation of the inner and outer coat layers, respectively (Zheng et al., 1988; Takamatsu et al., 1999; Abhyankar et al., 2013; Stelder et al., 2018). In Clostridia, a recently identified protein, CotL, has been deemed important for assembly of the coat, although its function is poorly understood (Alves Feliciano et al., 2019). Altogether, the endospore coat usually comprises multiple layers, each characterized by distinct morphogenic and structural proteins (McKenney et al., 2010; Stelder et al., 2018). While morphogenic proteins are relatively conserved, structural proteins can vary by organism and are tailored to its respective environmental niche (Abhyankar et al., 2013). Despite its thickness and a significant role in protection from various stressors, the coat is not dense enough to fully restrict movement of molecules, such as germinants, into the intramembrane space and core (Knudsen et al., 2016). Germination (Ger) receptors, are loaded from the mother cell during maturation and reside in the IsM. Lytic proteins involved in germination and degradation of the endospore coat and cortex are also embedded in the IsM. Finally, the proteinaceous coat also provides a mechanism for core expansion during rehydration through expansion of its layers (Sahin et al., 2012).

Some endospore forming species, such as *B. anthracis* and *A. longum*, have an additional outer layer composed of proteins, lipids and carbohydrates called the exosporium. The appearance of the exosporium differs by organism, can exhibit various thickness and includes appendage-like structures (Abhyankar et al., 2013; Pizarro-Guajardo et al., 2016). Although the exosporium provides a barrier for entry of large molecules, its exact functions are not known (Knudsen et al., 2016). Some proteins previously identified in the exosporia of *Bacillus* and *Clostridium* species could be relevant in pathogenesis, attachment or endospore dissemination (Abhyankar et al., 2013; Stewart, 2015; Pizarro-Guajardo et al., 2016).

On the other hand, the Actinobacterial exospore coat is characterized by a simpler architecture. For example, a thin spore coat has been observed on the surface of the spore wall of *Streptomyces* spores (Sexton and Tocheva, 2020). However, the composition and function of this coat in spore survival remain to be characterized. Some *Streptomyces* species produce elaborate coatings on the surface reminiscent of the exosporium produced by Firmicutes (Dietz and Mathews, 1970), likely in order to promote attachment and dispersal. It is unknown if these elaborate coats offer additional protection to the spore.

### Spore Release

Endospores are released through lysis of the mother cell. However, little is known about the regulation and dynamics of programmed death of the mother cell. Time-lapse fluorescence microscopy of *B. subtilis* reveals that the endospore is released from the cell pole by rupturing of the mother cell membrane at both poles, characterized by an intense signal from the membrane dye FM4-64 at the mother cell poles and then inside the cytosol (Hosoya et al., 2007). Simultaneously, decreased fluorescence from DNA dyes is observed in the mother cell. This suggests that lipid membranes become ruptured, and DNA gets degraded inside the mother cell prior to cell wall collapse and complete release of the endospore (Hosoya et al., 2007). Autolysins such as CwlB, CwlC, and CwlH, under the control of σ^K^, have been shown to play major roles in cell wall collapse and the subsequent lysis of the mother cell through PG degradation in *B. subtilis* (Smith and Foster, 1995; Nugroho et al., 1999). In diderm endospore formers, such as Negativicutes, this would likely be insufficient and would require additional players for degradation of the OM. Cryo-electron tomography imaging of the diderm endospore former *A. longum* reveals loosening of the mother cell OM in endospore maturation, suggesting that the OM likely “blebs off” during spore maturation (Tocheva et al., 2011). However, mother cell lysis is not well described outside the model organism *B. subtilis*.

In contrast, spore release occurs in a more direct manner for exospore formers. Exospores are released from the spore chain when the rodlet ultrastructure on the cell surface is mechanically disrupted (Di Berardo et al., 2008). This could be accomplished by biotic factors, such as passing insects, or abiotic factors, such as wind or ocean currents.

Endospores ultimately have enhanced resistance to a variety of environmental stressors compared to exospores (Table 1). This includes heat tolerance, where 60% of *Streptomyces* spores are viable after heating to 60°C for 10 min, but no spores are viable after heating to 80°C for 10 min (Kawamoto et al., 1981; Haiser et al., 2009). In contrast, endospores produced by *Bacillus* are well adapted to tolerate extended heat treatment. At 80°C in a humid environment, 50% of *Bacillus* spores remain viable after 30 h of exposure (Setlow, 2014). DPA, core dehydration, and heat shock proteins are proposed to protect the cell against damage from wet heat (Setlow, 2014). Endospores are also significantly more resistant to desiccation and UV damage compared to exospores (Table 1). Both desiccation and UV impact spore viability through DNA damage (Setlow, 2014), which suggests that DPA and SASPs found in endospores are a more robust mechanism for DNA protection than trehalose and the combination of nucleoid associated proteins found in exospores (Setlow, 2014).

## Germination

Germination stimuli are diverse, vary between species, and include free amino acids, PG molecules, organic acids, as well as chemicals specifically tailored to the ecological niche (e.g., bile salts for the intestinal pathogen *C. difficile*) (Bhattacharjee et al., 2016). In Firmicutes, germination receptors (Ger) residing in the IsM sense signals from the environment via interaction with small molecules (germinants) penetrating the spore cell wall. Ger type receptors are conserved across endospore formers, but show variability in the types of signals they receive, meaning that structure alone cannot be used to predict cognate germinants (Ross and Abel-Santos, 2010). Ger receptors form membrane-spanning complexes in the IsM and are often implicated in recognition of free amino acids (Ross and Abel-Santos, 2010). Preliminary investigations into the structure of Ger receptors reveal that they are similar to inner membrane-associated small molecule transporters and signal transducers, possibly forming clusters (Li et al., 2014, 2019). Small PG fragments produced during spore germination stimulate germination of *B. subtilis* spores by binding to PrkC, a Ser/Thr kinase containing a peptidoglycan binding PASTA domain (Shah et al., 2008). PrkC is conserved in Clostridia (Shah et al., 2008), so it may play a broad role in stimulating germination of spores produced by Firmicutes. In *B. subtilis*, PrkC is proposed to phosphorylate the elongation factors EF-G and EF-Tu to promote ribosome assembly and translation elongation (Shah et al., 2008; Absalon et al., 2009; Pompeo et al., 2012). It is likely that PrkC phosphorylates additional targets to promote germination, although these are yet to be defined. Additionally, Clostridia possess Csp-type receptors indirectly connected to the OsM via lipoproteins and coupled to the cortex lytic enzyme SleC, which is activated by cleavage upon recognition of germinant (Fimlaid et al., 2015a; Bhattacharjee et al., 2016; Kochan et al., 2018; Donnelly et al., 2020). Organisms such as *C. difficile* lack Ger-type receptors altogether and instead rely on Csp proteins, such as CspC (Bhattacharjee et al., 2016). Structural studies of Csp-type receptors reveal that they function as subtilisin-like proteases in activation of SleC, but the complete mechanism for signal recognition and integration remains unelucidated (Adams et al., 2013; Rohlfing et al., 2019).

Stimulants activate a signal cascade triggering germination. In *B. subtilis*, a cluster of proteins called the germinosome is hypothesized to mediate this process. The germinosome comprises germination receptors, scaffolding proteins and other components, such as the SpoVA protein involved in DPA transport (Griffiths et al., 2011). Fluorescence visualization studies reveal that the germinosome localizes at one pole of the dormant endospore, then disperses as germination progresses (Troiano et al., 2015; Breedijk et al., 2020; Wang et al., 2020). The germination cascade is then followed by degradation of the spore cell wall by lytic enzymes, increasing permeability and allowing Ca^2+^-DPA complexes and ions to escape the endospore in order to be replaced with water from the environment (Griffiths et al., 2011; Wang et al., 2015). Cortex hydrolysis in the Bacilli is mediated by the conserved enzymes SleB and CwlJ (Setlow, 2003). While many Clostridia similarly encode and utilize these enzymes, they also rely on an alternative mechanism of cortex lysis by the enzyme SleC activated by the Csp complex (Kochan et al., 2018; Shen et al., 2019). Mechanism for protein coat degradation remains to be investigated, but possibly involves OsM or coat-associated proteases which initiate thinning from within (Santo and Doi, 1974; Jagtap et al., 2006; Plomp et al., 2007). As the spore swells rapidly, rehydration and changes in core pH allow for resumption of metabolic activity (Setlow, 2003; Liang et al., 2014). Subsequently, DNA-binding SASPs become degraded and provide building blocks for biosynthesis, as well as allow for gene expression upon chromosome release (Jagtap et al., 2006; Swarge et al., 2020). Core mRNA, prepackaged in maturation, is also degraded in this manner and likely not used for protein expression (Setlow and Kornberg, 1970; Swarge et al., 2020). Transcriptomic and proteomic studies in *Bacillus* and *Clostridium* endospores reveal that the first genes to become expressed upon resumption of metabolic activity include those for transcription regulation, biosynthesis, DNA repair, and nutrient uptake pathways. Cell growth, elongation and cell wall remodeling become activated during the next stage (outgrowth). Cell division, motility and biofilm formation are activated last upon resumption of vegetative growth (Keijser et al., 2007; Bassi et al., 2013; Dembek et al., 2013; Swarge et al., 2020; Xing and Harper, 2020).

Upon endospore coat shedding, cortex degradation, and dissolution of the OsM, monoderm Firmicutes, such as *Bacillus*, emerge as cells surrounded by a cytoplasmic membrane and a layer of vegetative-like PG, termed the germ cell wall, pre-deposited under the cortex and serving as a scaffold for growth of additional PG (Figure 2J) (Tocheva et al., 2013; Sella et al., 2014). In contrast, germination and outgrowth in diderm bacteria, such as the Negativicute *A. longum*, require extensive cortex hydrolysis and the remodeling of their inner-membrane like OsM into a typical OM (containing lipopolysaccharide, LPS and β-barrel OMPs) (Figure 2K). The mechanism of this novel membrane remodeling is not known (Tocheva et al., 2011).

Similar to endospores, *Streptomyces* spores remain dormant in the environment until they are stimulated to germinate. *Streptomyces* species do not contain any known homologs of Ger receptors for signaling germination, and PASTA domain containing kinases which sense PG fragments do not function to stimulate germination (Sexton et al., 2020). Instead, divalent cations such as calcium are known to function as germinants (Eaton and Ensign, 1980), although the mechanism underlying this stimulation is unclear. Following the signal to initiate germination, the spore swells and switches from phase bright to phase dark, and outgrowth occurs. This stage relies on several of the macromolecules deposited in the spore during preparation for dormancy, including proteins and mRNAs used for protein synthesis (Strakova et al., 2013). Outgrowth relies on the activity of cell wall lytic enzymes to make space for new polar growth to occur (Sexton et al., 2020). The innermost layer of the spore wall is continuous with the new vegetative cell wall, and the coat and outer layer of the spore wall peel away from the germinating exospore (Figure 2L) (Sexton and Tocheva, 2020). Tip extension and branching is then used to produce the vegetative mycelium which colonizes the environment.

## Genetic Differences and Evolution

**FIGURE 3:**
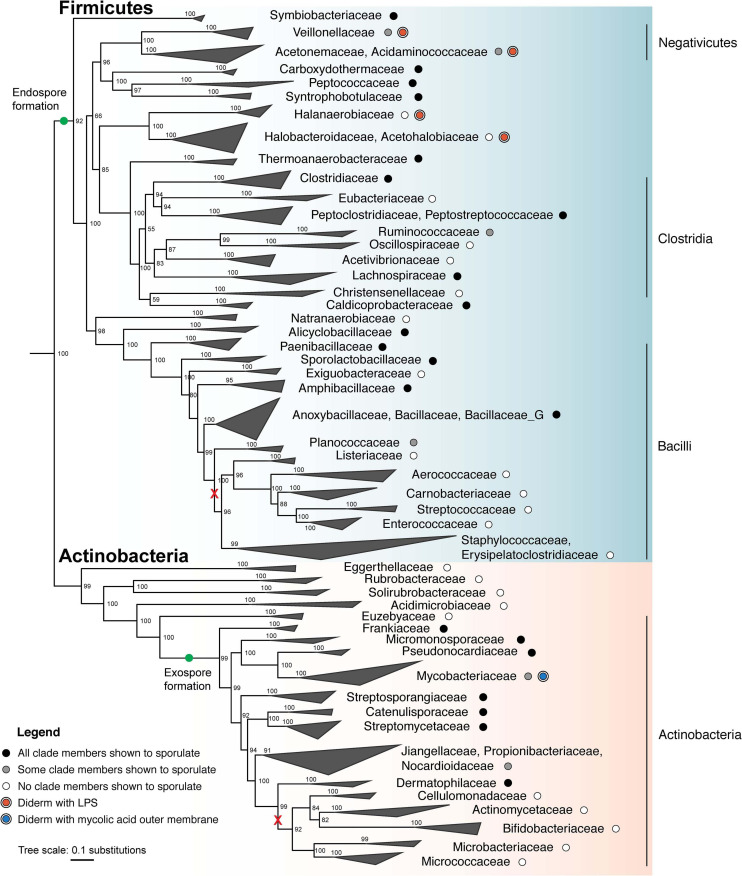
Maximum likelihood phylogeny of sporulating and non-sporulating Firmicutes and Actinobacteria. Tree was constructed using an alignment of 120 single-copy marker gene sequences from several hundred representative genomes from each phylum using GTDB-Tk (Parks et al., 2018; Chaumeil et al., 2019). Whole genome phylogeny was determined using the concatenated marker gene alignment and IQ-TREE (v. 2.0.3), with the substitution model LG + G4 and 1000 ultrafast bootstraps (Minh et al., 2020). The tree was subsequently down-sampled and collapsed to show major families. Several representative Cyanobacteria genomes served as an outgroup. Tree was visualized using ggtree (Yu, 2020). Green dots indicate that endospore formation was likely present in the last universal ancestor of all Firmicutes, whereas exospore formation appears to have evolved after phylum differentiation. Black and gray dots represent the demonstrated ability to sporulate in all members and some members, respectively, whereas white dots represent the lack of sporulation ability. Major losses involving multiple families are shown (red x). Clades which include diderm bacteria are indicated for possession of either an LPS-containing OM (red dots) or a mycolic acid-containing OM (blue dot). Classes, as defined by the GTDB, are labeled on the right.

With regards to classification of endospore formers, phylogeny remains a point of controversy. Historically, endospore formation genes have been shown to differ between two groups within Firmicutes: bacteria of the class Bacilli, and a large taxonomic group (hereby referred to as the Clostridia-like spore-formers) which initially comprised bacteria typically assigned to the class Clostridia but was later expanded to include other Firmicutes carrying homologous sporulation genes, such as the Negativicutes, Peptococcaceae, and Halanaerobiales. Although our analysis based on the current data from GTDB indicates otherwise (Figure 3), some studies propose placement of these taxonomic groups within the class Clostridia (Yutin and Galperin, 2013; Davidson et al., 2018). Despite the questionable taxonomic placement, spore-forming Negativicutes display higher similarity in sporulation genes and proteins of typical Clostridia than Bacilli, pointing to their common evolutionary origins (Ramos-Silva et al., 2019). Altogether, such evidence suggests that diversification of endospore formation machinery between the classes Bacilli and Clostridia occurred early in the evolutionary timeline, preceding the emergence of Negativicutes. However, because the spore-forming Negativicutes are not yet genetically tractable, functional studies of Clostridia-like sporulation machinery are limited to the canonical representatives of the class Clostridia.

Comparative genomics and functional studies reveal several trends in sporulation mechanisms of Bacilli and the Clostridia-like spore formers. As such, despite the overall conservation of Spo0A and the associated phosphorylation mechanisms for initiation of sporulation in both groups, members of the class Clostridia lack the intermediate proteins Spo0F, Spo0B, as well as the associated phosphatases bridging the histidine kinases to the master regulator (Galperin et al., 2012; Abecasis et al., 2013; Al-Hinai et al., 2015). Moreover, the regulation of gene expression in endospore formation and maturation by the sporulation-specific sigma factors, despite the same chronological progression, occurs in a semi-independent manner in Clostridia (Al-Hinai et al., 2015). In general, all sporulation-associated machinery, including those involved in cell division, DNA transport and compaction, cortex formation, the AA-AH complex, DPA synthesis, spore coat formation and germination, is conserved between all endospore formers (Galperin et al., 2012; Abecasis et al., 2013; Ramos-Silva et al., 2019). However, minor differences are observed with occurrence of supplementary pathways (such as DPA synthesis by EtfA, and Csp-mediated triggering of germination in the Clostridia-like spore formers; Orsburn et al., 2010; Rohlfing et al., 2019), replacement of individual components by their functional homologues (assembly of the spore coat base via SpoVM in *Bacillus* and SipL in *Clostridium*; Ribis et al., 2017), losses of intermediate components (modification of Spo0A via a phosphorelay system in Bacilli and a two-component system in Clostridia), and slight discrepancies in co-regulation and co-dependence of cellular processes (e.g., influence of deficiencies in spore coat assembly on cortex formation; Ebmeier et al., 2012; Benito de la Puebla et al., 2020).

Analysis of sporulation-associated genes also aids in elucidating the evolutionary history of Firmicutes as a phylum. Due to the extensive, multi-step nature of sporulation, horizontal transfer of sporulation genes would likely not be successful. The observation that endospore formation is widely spread throughout the phylum in both monoderm and diderm members indicates that the ability to sporulate was an ancient feature (Figure 3). Intriguingly, diderm Firmicutes possess an OM that is identical to that of classical Gram-negative bacteria of the phylum Proteobacteria. The recent discovery that two independent diderm lineages (Negativicutes and Halanaerobiales) exist in Firmicutes that share OM features with Proteobacteria, argues against horizontal gene transfer of OM genes and further points to a diderm ancestor of Firmicutes (Figure 3) (Megrian et al., 2020; Taib et al., 2020). Altogether, data show that the ability to sporulate and the presence of a true OM in Firmicutes were present in the last common ancestor of the phylum (Figure 3) (Tocheva et al., 2016). Subsequent loss of either sporulation or the OM could explain the observed diversity in modern species. For example, among the Negativicutes class, both spore-forming (e.g., *A. longum*) and non-spore forming (e.g., *Veillonella parvula*) diderm organisms have been described (Tocheva et al., 2011), indicating of loss of sporulation. Analogously, monoderm and diderm sporulators (*B. subtilis* and *A. longum*, respectively) have also been described, indicating OM loss in monoderm species. Gene losses in endospore and/or OM formation pathways along the evolutionary timeline would thus result in the diversity observed in Firmicutes today, yielding monoderm non-spore formers (e.g., *Lactobacillus* sp.), monoderm spore-formers (e.g., *B. subtilis*), diderm non-spore formers (e.g., *V. parvula*) and diderm spore-formers (e.g., *A. longum*).

Within the phylum Actinobacteria, sporulation is restricted to a single branch (class Actinobacteria, Figure 3) which evolved roughly 1.7 billion years ago. Because all sporulating Actinobacteria have evolved from a single lineage, it is thought that sporulation evolved once within the phylum, and speciation has resulted in the diversity of modes for producing exospores (green dot, Figure 3) (Chandra and Chater, 2014). While many of the regulatory networks controlling sporulation in the Actinobacteria are conserved, the genes within the regulons are not. This is likely due to the fact that sporulation within the Actinobacteria ranges from production of single exospores on vegetative mycelia to elaborate sporangia containing spores. As a result, the core set of genes responsible for exospore formation within the Actinobacteria is still poorly defined. However, *bldD* is conserved in all sporulating Actinobacteria, indicating that intracellular c-di-GMP levels govern the initiation of sporulation across the diverse modes of sporulation. *whiG* is not conserved outside of Actinobacteria which produce long chains of exospores (Chandra and Chater, 2014), suggesting that this level of regulation is unique to this specific type of exospore production. SsgB is conserved in most sporulating Actinobacteria, although its interacting partner SsgA is only found in the streptomycetes (Chandra and Chater, 2014). MreB is not conserved in all sporulating Actinobacteria, along with other members of the *mre* gene cluster including MreC, MreD, PBP2, and Sfr (Chandra and Chater, 2014), suggesting that there may be alternative mechanisms for synthesizing the spore wall. Finally, genes for trehalose biosynthesis are broadly conserved in all sporulating Actinobacteria, suggesting that all exospores produced by Actinobacteria use trehalose to conserve their cytoplasmic contents during dormancy. Notably, sporulation and filamentous growth are inextricably linked within the Actinobacteria, as all filamentous organisms produce exospores. Filamentous growth and sporulation have been lost independently several times throughout the Actinobacteria, such as in *Mycobacterium*, *Corynebacterium*, *Propionibacterium*, and *Rhodococcus*. The inability of these organisms to sporulate is correlated with a loss of several genes associated with exospore formation (Chandra and Chater, 2014). Importantly, the evolution of the mycobacterial OM occurred independently of the LPS-containing OM within Firmicutes (Vincent et al., 2018).

## Concluding Remarks

Despite numerous parallels between endospore and exospore formation, such as similar environmental stimuli and spore envelope architecture, it is clear that these processes have distinct regulatory, structural, and evolutionary origins, and thus represent an example of convergent evolution.

Nutrient limitation drives sporulation in both Firmicutes and Actinobacteria, and both endo- and exospore formation are controlled by a central transcription factor, albeit in distinct ways (activation by Spo0A for endospore formers, and repression by BldD for exospore formers). The response to yet-to-be-determined environmental cues impacts the DNA-binding ability of these regulators, triggering a downstream cascade of transcription and sigma factors, which sequentially direct the expression of genes required for sporulation. Both endo- and exospore formation involve cell division and chromosome segregation by homologous machinery – however, the results of these processes differ vastly between the two modes of sporulation (production and engulfment of a daughter cell in the Firmicutes, and simultaneous generation of multiple non-engulfing spores in Actinobacteria). Later stages of endo- and exospore formation, including cell envelope transformations and metabolic adaptations for initiation of dormancy, occur through superficially similar, but evolutionarily independent pathways. However, both Firmicutes and Actinobacteria utilize the mechanisms of cell wall thickening, DNA compaction and spore core dehydration for enhanced resilience under stressful environmental conditions. Finally, reintroduction of dormant spores into a more favorable environment results in germination in both endo- and exospore formers, but their distinct ecological niches, as well as the difference in overall cell cycles, necessitate utilization of vastly independent processes for resumption of vegetative growth.

## Author Contributions

PB, DS, MG, AH, and ET wrote the text and prepared the figures. All authors contributed to the article and approved the submitted version.

## Conflict of Interest

The authors declare that the research was conducted in the absence of any commercial or financial relationships that could be construed as a potential conflict of interest.
